# Retraction: The Use of Surrounding Rock Loosening Circle Theory Combined with Elastic-plastic Mechanics Calculation Method and Depth Learning in Roadway Support

**DOI:** 10.1371/journal.pone.0301148

**Published:** 2024-03-20

**Authors:** The PLOS ONE Editors

The *PLOS ONE* Editors retract this article [1] because it was identified as one of a series of submissions for which we have concerns about peer review integrity and similarities across articles. These concerns call into question the validity and provenance of the reported results. We regret that the issues were not identified prior to the article’s publication.

Prior to being notified about the above concerns, the corresponding author requested that this article be withdrawn because they felt that the research is not thorough enough and requires refinement.

QX, YL, and LZ agreed with the retraction. JL either did not respond directly or could not be reached.
